# Triple-seronegative myasthenia gravis: clinical and epidemiological characteristics

**DOI:** 10.1055/s-0044-1779052

**Published:** 2024-02-05

**Authors:** Paula Raquel do Vale Pascoal Rodrigues, Cláudia Suemi Kamoi Kay, Renata Dal-Pra Ducci, Marco Antonio Takashi Utiumi, Otto Jesus Hernandez Fustes, Lineu Cesar Werneck, Paulo José Lorenzoni, Rosana Herminia Scola

**Affiliations:** 1 Universidade Federal do Paraná, Hospital de Clínicas, Departamento de Clínica Médica, Serviço de Neurologia, Serviço de Doenças Neuromusculares, Curitiba PR, Brazil.; 2 Universidade Federal do Paraná, Programa de Pós-Graduação em Medicina Interna, Curitiba PR, Brazil.

**Keywords:** Myasthenia Gravis, Antibodies, Receptors, Cholinergic, Acetylcholine, Miastenia Gravis, Anticorpos, Receptores Colinérgicos, Acetilcolina

## Abstract

**Background** *Myasthenia gravis* (MG) is an autoimmune disease usually caused by antibodies against the acetylcholine receptor (AChR-Abs), muscle-specific tyrosine kinase (MuSK-Abs), or low-density lipoprotein receptor-related protein 4 (LRP4-Abs). However, there are MG patients who do not have these antibodies and are thus said to have triple-seronegative (triple-SN) MG.

**Objective** This study aims to describe the frequency and clinical and epidemiological characteristics of patients with triple-SN MG.

**Methods** This was a retrospective cross-sectional study carried out through the analysis of medical records. Descriptive and analytical statistical analysis was performed comparing subgroups of myasthenic patients, classified according to serological profile.

**Results** The sample population consisted of 93 MG patients: 85 were positive for antibodies, 80 (86%) with AChR-Abs, 5 (5.4%) with MuSK-Abs, and no MG patients with LRP4-Abs. Eight patients (8.6%) had triple-SN MG; they had a median age at disease onset of 30 years (21-45). Their most common initial symptoms were ptosis, diplopia, and generalized weakness. Most patients presented with mild symptoms at their last visit, reflecting a median MG composite scale score of 4 (0-6), and 75% of patients had an adequate response to treatment.

**Conclusion** Our study showed a low frequency of triple-SN MG in Brazilian MG patients. Triple-SN MG was predominant in females, who presented with ptosis, diplopia, and generalized weakness, and most patients had an adequate response to immunosuppressive treatment. There was no significant difference between triple-SN MG and the other subgroups.

## Resumo

**Antecedentes** A *Miastenia gravis* (MG) é uma desordem autoimune geralmente causada por anticorpos antirreceptores de acetilcolina (anti-RACh), tirosina quinase músculo-específica (anti-MuSK) ou proteína 4 relacionada ao receptor de lipoproteína de baixa densidade (anti-LRP4). No entanto, em uma parcela dos pacientes, nenhum destes três anticorpos pôde ser detectado, sendo estes casos denominados “triplo-soronegativos”.

**Objetivo** Descrever a frequência, bem como as características clínicas e epidemiológicas dos pacientes com MG triplo-soronegativa.

**Métodos** Consiste em um estudo transversal e restrospectivo, realizado através da análise de prontuários médicos. Foi realizada análise estatística descritiva e analítica entre os subgrupos de pacientes, classificados de acordo com o perfil sorológico.

**Resultados** A população consistiu de 93 pacientes com MG: 85 pacientes apresentavam positividade para anticorpos, sendo 80 (86%) com anticorpos anti-RACh, cinco (5,4%) com anti-MuSK, e não foram encontrados pacientes com anti-LRP4. Oito (8,6%) eram pacientes triplo-soronegativos, que apresentaram idade média de início da doença de 30 anos (21-45), e com sintomas iniciais mais comuns de ptose, diplopia e fraqueza generalizada. 75% dos pacientes triplo-soronegativos apresentaram resposta adequada ao tratamento.

**Conclusão** O estudo demonstrou uma baixa frequência da pacientes com MG triplo-soronegativa na população brasileira. A MG triplo-soronegativa foi predominante nas mulheres, que se apresentaram com ptose, diplopia ou fraqueza generalizada, e a maioria dos pacientes apresentou resposta adequada ao tratamento imunossupressor. Não houve diferença significativa entre a MG triplo-soronegativa e os demais subgrupos.

## INTRODUCTION

Myasthenia gravis (MG) is an autoimmune disorder characterized by transmission failure at the neuromuscular junction (NMJ), which results in fluctuating muscle weakness and fatigability. 1 2

Antibodies against acetylcholine receptor (AChR-Abs) on the postsynaptic membrane of the NMJ can be detected in 80-85% of MG patients. In the remaining MG patients, antibodies against other components of the postsynaptic membrane can be found, such as muscle-specific tyrosine kinase antibody (MuSK-Abs) in 5% of patients 3 4 5 6 and anti-low-density lipoprotein receptor-related protein 4 antibody (LRP4-Abs) with a variable incidence. 2 6 7

Even so, there are MG cases without autoantibodies against AChR, MuSK, or LRP4, that are called triple-seronegative (triple-SN) MG. 8 It can be speculated that the lack of autoantibody reactivity in these patients is due to the low sensitivity of the available tests or the presence of antigens not yet identified. 8 9

However, few studies have characterized individuals with triple-SN MG, and, to the best of our knowledge, there are none with Brazilian patients. This study aims to describe patients with triple-SN MG followed up in a tertiary care hospital in southern Brazil and compare them between groups with different serological profiles.

## METHODS

This was a cross-sectional study carried out through the retrospective analysis of clinical, treatment, and laboratory data and electrophysiological features of MG patients seen in a single neuromuscular center at the Hospital de Clínicas da Universidade Federal do Paraná (Curitiba, Brazil) between 2019 and 2021. The local Human Research Ethics Committee approved the study, which was conducted in accordance with ethical principles, CAAE-91509518.0.0000.0096.

Patients with possible MG were selected based on clinical findings, among these, we included patients who fulfilled clinical (fluctuating muscle weakness with fatigue), electrophysiological (repetitive nerve stimulation -RNS- with an abnormal decrement of compound muscle action potential greater than 10%) 10 or laboratory (detection of serum autoantibodies against AChR, MuSK or LRP4) diagnostic criteria for MG. Patients who satisfied both clinical and electrophysiological criteria but lacked antibodies against AChR, MuSK, and LRP4 were categorized as having triple-SN MG. 8 9

We excluded patients who did not have a serum antibody dosage performed, who did not undergo clinical follow-up, and those who had genetic analysis confirming other neuromuscular disorders (e.g., congenital myasthenic syndrome) conducted using a target next-generation sequencing (NGS) panel.

Genetic analyses of triple-seronegative MG patients were carried out using sequence analysis and deletion/duplication testing (NGS) of genes associated with neuromuscular disorders (including those associated with congenital myasthenic syndrome).

AChR-Abs were analyzed through radioimmunoprecipitation assay (RIPA) or enzyme-linked immunosorbent assay (ELISA). For MuSK-Abs and LRP4-Abs, RIPA and immunocytochemical analysis were used.

The MG patients were grouped according to their antibody status as follows:

- MG with AChR antibodies (AChR-Abs);
- MG with anti-MuSK antibody (MuSK-Abs);
- MG with anti-LRP4 antibody;
- Triple-SN MG.

The epidemiological and clinical data collected at the last medical evaluation included age at disease onset, symptoms, distribution of weakness (generalized and/or ocular), bulbar symptoms (characterized by dysphagia, dysphonia, and dysarthria), clinical status by MG composite scale (MGC), 11 disease therapy, duration of corticosteroid therapy, treatment outcome, thymectomy, and thymus histology.

The MG patients were classified as having an adequate, partial, or refractory response to treatment:

- adequate response was used for patients who had minimal manifestation status or better or a 3-point fall in MGC score;
- partial response included those lacking the criteria for an adequate and refractory response; and
- refractory response occurred in MG patients who did not improve (or even worsened) with corticosteroids and at least two other immunosuppressive medications, presenting with persistent symptoms or side effects that limited functioning. 12

The duration of corticosteroid use was classified as up to one year, between one and five years, over five years, or not informed.

The data were analyzed using descriptive statistical methods. The qualitative variables are expressed as absolute and relative frequencies, and the independence between groups was analyzed with Fisher's exact test. Post hoc comparisons were carried out using simple regression models with Firth's method. 13 The quantitative variables are given as the median and interquartile range (first quartile, third quartile), and the Kruskal-Wallis test was employed to compare them. Descriptive logistic regression models were fitted with a backward elimination algorithm. All analyses were conducted in R version 4.0.2, always considering a significance level of 5%.

## RESULTS

**Figure 1:**
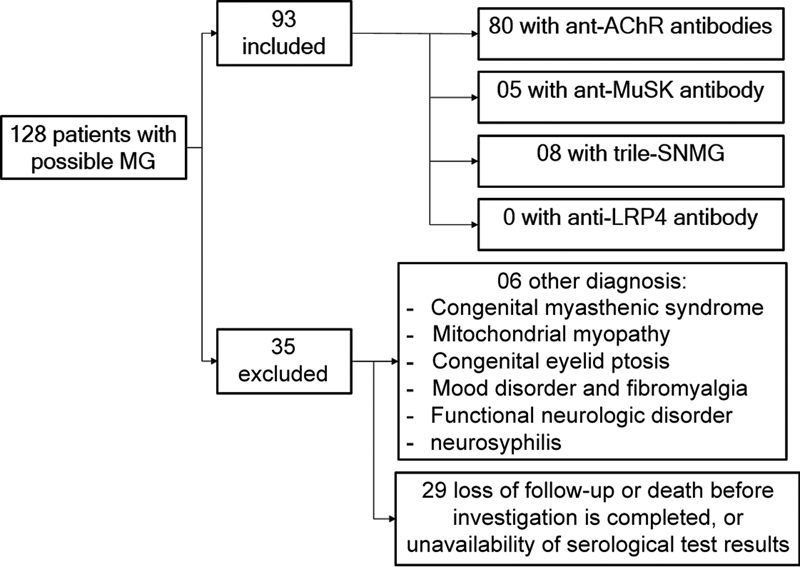
Flow diagram of included patients and antibody profile. Abbreviations: AChR, acetylcholine receptor; LRP4, low-density lipoprotein receptor-related protein 4; MG, myasthenia gravis; MuSK, muscle-specific tyrosine; SNMG, seronegative myasthenia gravis.

A total of 128 patients diagnosed with possible MG were found. Of these 93 individuals were included: 80 patients with AChR-Abs (86%), 5 patients with MuSK-Abs (5.3%), and 8 triple-SN MG (8.6%). None of these patients had a diagnosis of congenital myasthenic syndrome at genetic analysis. No patient with LRP4-Abs was found in this population ( Figure 1 ).

The median age of disease onset was 28 years (22-43) in AChR-Abs MG patients, 38 years (24-48) in MuSK-Abs MG subjects, and 30 years (21-45) in triple-seronegative individuals (p = 0.947). Female patients were found more frequently in all groups, representing 70% in AChR-Abs MG, 100% in MuSK-Abs MG, and 75% in triple-SN MG (p = 0.462). There was no significant difference regarding sex or age of MG onset between groups ( Table 1 ).

**Table 1:** Demographics and clinical findings according to the antibody status.

		AChR-Abs MG Number of cases (%)	MuSK-Abs MG Number of cases (%)	Triple-seronegative MG Number of cases (%)	p**
**Total**		80 (86)	5 (5.4)	8 (8.6)	
**Age at disease onset (years)**		29 (22-43)	38 (24-48)	30 (21-45)	0.947**
**Male:Female**		24 (30): 56 (70)	0: 5 (100)	2 (25): 6 (75)	0.462***
**Weakness distribution**	Ocular	7 (9)	0 (0)	2 (25)	0.235***
	Generalized	73 (91)	5 (100)	6 (75)	
Presenting symptoms*	Ptosis	37 (46)	1 (20)	4 (50)	0.600***
	Diplopia	29 (36)	2 (40)	3 (38)	1.000***
	Generalized weakness	28 (35)	0 (0)	2 (25)	0.269***
	Dyphagia/dysphonia	11 (14)	2 (40)	0 (0)	0.163***
	Cervical weakness	1 (1)	1 (20)	1 (13)	0.050***
	Limb weakness	9 (11)	0 (0)	1 (13)	1.000***
	Chewing/facial weakness	7 (9)	0 (0)	0 (0)	1.000***
**Bulbar symptoms**		57 (71)	5 (100)	5 (63)	0.404***
**MG Composite Scale**		3 (1-7)	1 (0-8)	4 (0-6)	0.746**

Abbreviations: AChR, acetylcholine receptor; MG, myasthenia gravis; MuSK, muscle- specific tyrosine kinase. Notes: All quantitative variables are summarized as absolute frequency (relative frequency, %), while the quantitative variables are summarized as median (first quartile – third quartile).

Notes: *Since more than one manifestation is possible at presentation, the total sum could be>100%; **Kruskal-Wallis; ***Two-tailed Fisher's exact test.

The most common symptoms at disease onset are summarized in Table 1 . These symptoms did not differ significantly between groups, except for cervical weakness, which was higher in the MG MuSK-Abs subgroup than in other subgroups (p = 0.05) and was associated with an OR of 17.7 (95% CI 1.25-255.9) for MuSK-Abs MG compared to AChR-Abs MG. In addition, the occurrence of bulbar symptoms was more frequent in MuSK-Abs MG cases (100%) but did not reach statistical significance (p = 0.163).

Regarding the distribution of weakness, the generalized form was the most common in all groups (p = 0.235) ( Table 1 ).

The median MG composite score was 3 points (3-7) in the AChR-Abs MG group, 1 point (0-8) in the MuSK-Abs MG group, and 4 points (0-6) in the triple-SN MG group (p = 0.746) ( Table 1 ).

Abnormal RNS tests were found in 88% of AChR-Abs MG patients and all MG MuSK-Abs and triple-SN MG patients (p = 0.77).

Table 2 summarizes the key information about MG treatment and thymus pathology.

**Table 2:** Treatment response and thymus pathology.

		AChR-Abs MG Number of cases (%)	MuSK-Abs MG Number of cases (%)	Triple-seronegative MG Number of cases (%)	P+
**Treatment response**	Adequate	51 (64)	3 (60)	6 (75)	1.000
	Partial	26 (32)	2 (40)	2 (25)	
	Refractory	3 (4)	0 (0)	0 (0)	
**Thymectomy**		23 (29)	2 (40)	3 (38)	0.869
**Thymus histology**	Hyperplasia	4 (17)	1 (50)	0 (0)	0.844
	Thymoma	4 (17)	0 (0)	1 (33)	
	Normal	2 (9)	0 (0)	0 (0)	
	Unknown	13 (57)	1 (50)	2 (67)	

Abbreviations: AChR, acetylcholine receptor; MG, myasthenia gravis; MuSK, muscle- specific tyrosine kinase.

Note: All variables are summarized as absolute frequency (relative frequency,%). The independence between groups was analyzed using the two-tailed Fisher's exact test.

Most patients had an adequate response to standard therapies for MG in all subgroups. However, MuSK-Abs MG patients were more likely to have a partial response, and refractory patients were only found in the AChR-Abs MG group ( Table 2 ). The difference was not statistically significant (p = 1.000). The duration of corticosteroid treatments was similar between the groups, with most patients having used them for one to five years (p = 0.341).

The triple-SN MG patients were treated with symptomatic treatment with acetylcholinesterase inhibitors, associated with corticosteroids in 25% of patients, and azathioprine plus corticosteroids in 75% of patients. Most triple-SN MG patients (75%) were under immunosuppressive therapy at the time of antibody analysis. One patient evolved with remission and is currently off medication, and only one triple-SN MG patient had a myasthenic exacerbation, although, without the need for ventilatory support or orotracheal intubation.

A thymectomy was performed in 29% of AChR-Abs MG patients, 25% of MuSK-Abs MG patients, and 38% of triple-SN-MG patients (p = 0.869). Thymoma and thymic hyperplasia were found in 34% of AChR-Abs MG patients, while 9% of them had a normal thymus according to the pathological study. One patient with triple-SN MG had thymoma, and another with MuSK-Abs MG had thymic hyperplasia, but the prevalence of thymus disease was not different among the subgroups.

Approximately 14% of MG patients had other autoimmune diseases, such as Grave's disease, neuromyelitis optica spectrum disorder, Sjogren's syndrome, and sclerosing cholangitis. Although MuSK-Abs MG patients were more likely to present with these comorbidities (40%) than those with AChR-Abs MG (13%) and triple-SN MG (13%), the differences were not statistically significant (p = 0.214).

The most common comorbidities in triple-SN MG were endocrinological (diabetes, dyslipidemia, osteoporosis, obesity) in 26% of patients. Other autoimmune diseases were found in 13% (Grave's disease) and psychiatric diseases in 13% (mood disorders).

## DISCUSSION

In this study, we found a prevalence of triple-SN-MG patients of 8.6%, which is slightly lower than that reported in previous studies. 2 8 The MuSK-Abs MG and AChR-Abs MG subgroups accounted for 5.4% and 86% of the sample, respectively. There were no LRP4-Abs-positive patients in our sample, which is similar to that described by Cortés-Vicente et al. 14 Other reports have shown a highly variable prevalence of the LRP4-Abs MG subtype in different populations, from 1-5% of all patients with MG to 50% of the double-SNMG cases. 15 16 17

The lack of patients with LRP4-Abs might be due to the low sensitivity of the laboratory method used, the sample size, or the local ethnic and genetic backgrounds. The mixture of European, African, and indigenous genes in the Brazilian population may explain this difference and may reflect distinct genetic profiles in the Brazilian MG population. This was described by Werneck et al. 18 in a study of multiple sclerosis patients and controls HLA. However, in a large study of 800 patients with MG from 10 different countries, Zisimopoulou et al. 6 did not identify an obvious pattern due to geographical variation. Moreover, Rivner et al. 19 included minority patients in their study and found no differences between races, although lower incidences have been reported in Asian populations.

The age at disease onset and the proportion of women were similar among the AChR-Abs MG, MuSK-Abs MG, and triple-SN-MG subgroups, similar to what was described by Deymeer et al. 20 However, in a previous study of a South African cohort, individuals with MuSK-Abs MG manifested the disease at a younger age. 21

In our cohort, the most common presenting symptoms were ptosis, diplopia, and generalized weakness in the AChR-Abs and triple-SN-MG-Abs groups and ptosis, diplopia, dysphagia, and dysphonia in the MuSK-Abs MG group. These findings agree with previous reports that ocular symptoms are the most common presentation in myasthenic patients, with rapid progression to bulbar involvement in MuSK-Abs MG patients. 22 23 The initial manifestation of myasthenia with cervical weakness differed between groups (p = 0.05), being more associated with MuSK-Abs MG than with AChR-Abs MG. In a large series of 53 MuSK-Abs-positive MG patients, cervical weakness was reported in 92% of the patients. 24 In addition, weakness of the neck extensor muscles, which may present as head drop, can be an early symptom and should raise concerns specific to MuSK-Abs MG. In AChR-Abs MG, weakness of the neck flexor muscles is more typical. 25 26

Although there was no significant difference in the prevalence of bulbar symptoms between the groups, there was a trend toward a higher frequency of symptoms in MuSK-Abs MG than in AChR-Abs MG and triple-seronegative MG, as previously reported. 23 26 According to Rodolico et al., 27 bulbar impairment has been found in up to 80% of patients with MuSK-Abs MG.

All triple-SN-MG and MuSK-Abs MG patients had an abnormal response on the RNS test, while 88% of AChR-Abs MG patients did so. According to Rodolico et al., 27 the sensitivity of RNS seems to be lower in MuSK-Abs MG than in AChR-Abs MG, especially when performed on distal limb muscles, and it is possible to increase to 75-85% by testing proximal muscles, in particular the facial muscles. Since RNS reflects the integrity of NMJ transmission, a decrement is more often observed in clinically weak muscles. 10

Most triple-SN-MG patients had low scores on the MGC scale, and adequate treatment response, with no refractory patients in this group. Hong et al. 28 found that 90% of SNMG patients had a mild disease with severity I or II according to the Myasthenia Gravis Foundation of America (MGFA), and a higher proportion did not receive any immunosuppressive treatment compared with antibody-positive patients. Although there were no LRP4 MG-Abs patients in our cohort, previous studies found that these patients often presented with isolated ocular muscle weakness and mild severity (MGFA I-II). 29

Autoimmune overlap is well recognized in MG patients, but it seems to vary between its subgroups 30 and was found in 18.2% of triple-SN-MG patients in our study population. The overall frequency of autoimmune comorbidity in our MG patients was 14%, as in a previous report. 31 32

While the statistical analysis did not reveal any statistically significant differences in the evaluated clinical and epidemiological characteristics, trends were observed in the clinical presentation, disease severity, and response to therapy, suggesting a resemblance between triple-SN MG and AChR-Abs MG. Cortés-Vicente et al. 14 reported that double-SNMG (for MuSK-Abs and AChR-Abs) MG subjects were similar to those with AChR-Abs MG in terms of clinical characteristics and response to treatment.

However, triple-SN MG is possibly more clinically heterogeneous, due to a wide range of pathogenic mechanisms involved in these cases. 2 28 The available evidence suggests that the underlying mechanism is still autoimmune, given the response to immunosuppression, the lack of family history, and genetic alterations, the animal models with transfer of patient plasma or immunoglobulin G (IgG) causing neuromuscular transmission defects in rats 17 33 and the presence of complement deposits on the end plate of the skeletal muscle indicating complement activation via the classical IgG1-mediated pathway. 34

Furthermore, there are reports of antibodies against other targets, whose pathogenic mechanisms have yet to be proven, such as colQ, agrin, kv1.4 voltage-dependent potassium channel, and cortactin. 35 36 37 38 Moreover, some authors have identified clustered AChR-Abs that might have low affinity when conventional laboratory methods are employed. 9 39 Finally, although antibodies against muscle proteins such as titin and ryanodine may be used as markers of disease severity and the presence of thymoma, they play no role in the pathogenesis of MG. 32

In conclusion, in the study population, triple-SN MG showed a female predominance, and the most frequent initial symptoms were ptosis, diplopia, and generalized weakness. The majority of patients presented mild symptoms and had adequate responses to treatment.

The lack of positivity for antibodies in the triple-SN-MG subgroup may reflect the low prevalence of LRP4 patients in the Brazilian population, the low sensitivity of the laboratory assays used, or also a portion of patients with antigens not yet described or non-antibody mechanisms.

In the future, we believe that studies with larger sample sizes, using more sensitive laboratory assays, and including patients naive to immunosuppressive treatments, are needed for better characterization of triple-SN MG.
